# The Effect of Individual and Neighbourhood Socioeconomic Status on Diabetes Mellitus Survival in Working Age Patients in Taiwan

**DOI:** 10.1371/journal.pone.0169550

**Published:** 2017-01-12

**Authors:** Shih-Hsien Yang, Su-Feng Chen, Shin Nieh, Chia-Lin Liu, Yaoh-Shiang Lin, Ching-Chih Lee, Fu-Huang Lin

**Affiliations:** 1 Graduate Institute of Medical Sciences, National Defense Medical Center, Taipei, Taiwan; 2 Department of Medical Administration Office, National Defense Medical Center & Tri-Service General Hospital Beitou Branch, Taipei, Taiwan; 3 Department of Dental Hygiene, China Medical University, Taichung, Taiwan; 4 Department of Pathology, National Defense Medical Center & Tri-Service General Hospital, Taipei, Taiwan; 5 Department of Otorhinolaryngology, Head and Neck Surgery, Kaohsiung Veterans General Hospital, Kaohsiung, Taiwan; 6 Department of Otolaryngology, Head and Neck Surgery, Tri-Service General Hospital, Taipei, Taiwan; 7 School of Medicine, National Defense Medical Center, Taipei, Taiwan; 8 School of Public Health, National Defense Medical Center, Taipei, Taiwan; Taipei Medical University College of Medicine, TAIWAN

## Abstract

**Purpose:**

Diabetes mellitus (DM) is a global pandemic metabolic disorder. In recent years, the amount of medical resources required for the treatment of diabetes has increased as diabetes rates have gradually risen. The combined effects of individual and neighbourhood socio-economic status (SES) on DM survival rates are still not clear, especially in patients of working age. In this paper, we aim to analyze the combined effects of neighbourhood and individual SES on DM survival rates in patients of working age in Taiwan.

**Methods:**

The study of 23,781 people who were diagnosed with DM by using population—based study between 2002 and 2006. Each sample was followed up for 4 years or as a sensor case. We defined Individual SES and neighbourhood SES by each patient’s job category and household income which characterized as advantaged or disadvantaged. Then we compared the survival rates by SES group used Cox proportional hazards model for adjust risk factors.

**Results:**

The 4-year overall survival rates of diabetic patients were worst for those with low individual SES who living in advantaged neighbourhoods. After adjustment for patient characteristics, DM patients with high individual SES living in disadvantaged neighbourhoods had the same risk of mortality as those patients with high individual SES living in advantaged neighbourhoods (hazard ratio: 1.11; 95% confidence interval [CI]: 0.81–1.51). The study found that DM patients with low individual SES who live in disadvantaged areas had a greater risk of mortality than those with high SES (odds ratio: 2.57; 95% CI: 2.04–3.24). There were significant differences in survival rates between patients with high individual SES and patients with low individual SES. In contrast, the results did not statistically significant differences in survival rates between advantaged and disadvantaged neighbourhood SES groups.

**Conclusion:**

DM patients with low individual SES had the worst survival rate, regardless of whether they were living in a high or low SES neighbourhood area. The competitive cause of death, i.e., the fact that complications, rather than DM itself, are often the cause of death, may be the reason for the inverse relationship found between the effects of individual SES and neighbourhood SES on DM survival. We conclude that the socio-economic gradient in survival among DM patients may be the result of differences in access to medical treatment and attributes related to individual SES.

## Introduction

Diabetes mellitus (DM) is a global pandemic metabolic disorder. Its prevalence varies widely around the world, ranging from less than 1% of the population in some countries to more than 50% in others. DM is the most common disease resulting from insulin dysfunction, which results in a state of hyperglycaemia. There are two major primary types of DM: insulin-dependent DM and non-insulin-dependent DM. The prevalence of DM is increasing. The World Health Organization estimated that around 25.8 million people in the United States and more than 330 million people worldwide suffer from DM.[1, 2]

Diabetes is the seventh disease cause of death in the world, and over 90% of them are type 2 DM, which could be improved through behavioural modification, including improved nutrition and increased physical activity.[3–6] Factors that influence the survival rate of DM include socio-economic status (SES), age, gender, lifestyle, and other environmental factors. Estimated 1.5 million people died in the world in 2012 were directly caused by diabetes, and more than 80% of the deaths occurred in low- and middle-income countries, and the global prevalence was estimated to be 9% among adults until 2014.[7] About 50% of people with diabetes died from cardiovascular disease in a multinational study.[8] Diabetes can caused limb amputation and diabetic retinopathy and the death rate was at least two time as much as that of health person.[9]^,^[10]

According to the International Diabetes Federation (IDF), the prevalence of diabetes in the worldwide adult population (4.1 billion people aged 20–79 years in 2007) was 6.0%, which is equivalent to 246 million people. The IDF predicts that global diabetes will increase to 7.3% by 2025, which is equivalent to 380 million people, and that diabetes prevalence will grow by 101.7% as India, China, and the United States suffer from the diabetes pandemic.[11] In a study from 1996 to 2000, we found that the prevalence of diabetes in Taiwan increased over time and affected more women than men. In 1996, the prevalence of diabetes in men was 2.7% and in women 3.5%; by 2000, the prevalence for men had increased to 3.6% compared with 4.5% for women.[12] The prevalence of diabetes in Taiwan in 2004 was 5%, which is equivalent to 1.2 million people with diabetes. As prevalence is based on people seeking medical treatment of diabetes, which is estimated to account for 46–69% of all people with diabetes, the actual number of people with diabetes may be even higher.[13]

Diabetes can result in many complications, including blindness, amputations, and kidney failure, and in death, especially because of its relationship with cardiovascular disease. Treatment needs to focus beyond blood sugar control because there are other risk factors for cardiovascular disease (e.g., dyslipidemia, hyperinsulinemia, hypertension, and obesity) that must also be controlled. Another problem is noteworthy, i.e., the economic burden of diabetes. The annual cost of medical care has increased, with higher complications in mainland China and Australia.[14]^,^[15]

In Taiwan, the amount of medical resources required for the treatment of diabetes has gradually increased over recent years. According to the Taiwan Department of Health’s *National Health Insurance Health Statistics Annual*, medical costs for patients with diabetes as their primary diagnosis rose from 2001 to 2006, and the average cost was 4.3 times higher than nondiabetic patients, and outpatients were higher than those in inpatients. [16]

In 2000, diabetes and hospitalization expenses accounted for 13% of the total cost of hospitalization in Taiwan. Infectious diseases accounted for the majority of disease diagnoses in hospital diabetes inpatients, which was followed by cerebrovascular disease and chronic ischemic heart disease. The diabetes inpatient mortality rate was nearly double that of hospitalized non-diabetic patients. Data from a 3-year national analysis in Taiwan showed that the hospitalization expenses associated with diabetes are very high and that they have increased annually. There are issues related to limited distribution of medical resources and how best to assist ethnic groups who have high rates of diabetes. There is still much room for improvement in these areas. The combined effects of individual and neighbourhood SES on DM survival rates is still not clear, especially in patients of working age. Therefore, we have designed a population-based study using data from the Taiwan National Health Insurance Administration to analysis the combined effects of neighbourhood and individual SES on DM survival rates in patients of working age.

## Methods

### Ethics statement

The study was approved by the Institutional Review Board of Dalin Tzu Chi Hospital, Buddhist Tzu Chi Medical Foundation, Chiayi, Taiwan. The requirement for written informed consent was waived because all data were de-identified prior to analysis.

### Database

This dataset, which has been operating since 1995, is based on Taiwan’s National Health Insurance Program, organized and managed by the National Health Research Institutes in Taiwan. The plan covers about 97% of the medical providers and 99% of Taiwan residents. The study data were collected from 2002 to 2006 Taiwan’s National Health Insurance Research Database. Everyone who is interested in this dataset could access the NHIRD through the http://nhird.nhri.org.tw/ web link. The definition of working age is based on the Labour Standards Act of Taiwan, amended in 2011, under which the maximum age of retirement is 65 years.[17]

Our cohort study consists of incidental DM patients (based on the International Classification of Diseases (ICD), Ninth Revision, Clinical Modification [ICD-9-CM] code 250) in Taiwan who underwent treatment in a hospital for their disease at any time between 2002 and 2006.

### Measurement

The four-year survival rate is the key dependent variable of profits. There are specific reasons for the restricted use of registry data, but this was mainly because it did not provide useful cause-specific survival variables. In this study, we used a clinical morbidity index for use with ICD-9-CM administrative databases, but there were no significant difference in survival models for all-cause mortality.[18]

We designed individual and neighbourhood SES on survival as the main independent variables of this study. The survival of patients with diabetes was achieved by placing their 2002 to 2006 mortality data with claims data that indicated their first treatment for DM during the 4 years prior to the end of the study or death. Using the above information, we can calculate the DM survival rate. Patient characteristics included age, geographic region, gender, co-morbidities, and Urbanization. His presence in the co-morbidities was based on the modified Charlson’s co-morbidity index score (CCIS), a widely accepted measure for risk adjustment in administrative claims datasets.[19]

### Individual-level measures

In this study, we used the enrollee category, which defines a person’s workplace, as a proxy for individual SES, following validation of the use of this proxy by a previous study.[20] Subjects were divided into three groups: 1) high SES, comprising civil servants, employees of privately owned institutions or full-time, or regularly paid personnel with a government affiliation; 2) moderate SES, defined as members of the farmers’ or fishermen’s associations, and self-employed individuals, other employees; and 3) low SES, the definition for veterans, families of those unemployed, and alternative service draftees.

### Neighbourhood-level SES

Neighbourhood SES is a contextual factor of the 2001 census report based on the average family income and percentage of households in Taiwan. In that census, neighbourhood household income of the township, per capita income, which was determined by the Taiwan Ministry of Finance announced on the basis of the 2001 tax statistics.[21] The advantaged or disadvantaged neighbourhoods were sorted according to their median values: advantaged neighbourhoods had higher-than-median household incomes and disadvantaged neighbourhoods had lower-than-median household incomes.

### Other variables

We used population density, the percentage of residents with college level or higher education, the percentage of residents, residents of agriculture workers, number of physicians per 100,000 residents, urbanization level of residential divided into seven levels.[22] Urban areas were divided into level 1, the suburbs areas were divided into levels 2 and 3, and rural areas were divided into levels 4 to 7.

The study used accreditation level to distinguish the hospital as a medical centre, a regional hospital, or a district hospital. The geographical areas were recorded for the northern, central, southern, and Eastern Taiwan.

### Statistical analysis

The study of statistical operations were made using SPSS (version 15; SPSS Inc., Chicago, IL). Pearson’s chi-square test was used for categorical variables (level of urbanization, gender, category, and geographic region of residence) and characteristics of the hospital (ownership, teaching level, and workload). Continuous variables were analysed using one-way analysis of variance.

Cumulative 4 year survival rate and survival curve for each cohort were compared by log-rank test. The survival curves of the individual and neighbourhood SES were stratified by the overall mortality rate as an event variable.

Cox proportional hazards regression model was used to compare the results of different SES categories and after adjusting for patient characteristics (gender, age, urbanization, CCIS, and area of residence) and hospital characteristics (medical centre, district, and regional). Diabetes patients with low individual SES from disadvantaged communities were taken as the reference group. A two-sided p-value (*p* < 0.05) was considered statistically significant.

## Results

### Demographic data and SES characteristics

Table 1 shows the distribution of demographic and social variables, and how the variables differ depending on SES for all 23,871 DM patients. Compared to those with high individual SES were more likely to lower individual SES in the case of CCIS. There were statistically significant differences in age, gender, co-morbidities, geographic regions, and neighbourhood SES between the individual SES groups.

**Table 1:** Baseline characteristics, n = 23,871.

	Low SES	Moderate SES	High SES	*P value*
	*n* (%)	*n* (%)	*n* (%)	
Total	8769(100)	10325(100)	4777(100)	
Age (Mean ± SD)	65±14	65±12	56±9	<0.001
Gender				<0.001
Female	3806(43.4)	5643(54.7)	1569(32.8)	
Male	4963(56.6)	4682(45.3)	3208(67.2)	
CCIS (Mean ± SD)	2.3±1.7	2.2±1.7	1.9±1.5	<0.001
1	4053(46.2)	4792(46.4)	2773(58.0)	
2	1639(18.7)	2010(19.5)	723(15.1)	
≥3	3077(35.1)	3523(34.1)	1281(26.8)	
Neighbourhood SES				<0.001
Disadvantaged	3794(43.3)	7304(70.7)	1848(38.7)	
Advantaged	4975(56.7)	3021(29.3)	2929(61.3)	
Geographic Region				<0.001
Northern	4396(50.1)	3106(30.1)	2526(52.9)	
Central	1511(17.2)	2215(21.5)	782(16.4)	
Southern	2571(29.3)	4596(44.5)	1347(28.8)	
Eastern	291(3.3)	408(4.0)	122(2.6)	
Hospital characteristics Teaching level				<0.001
Medical center	2448(27.9)	2231(21.6)	1423(29.8)	
Regional	2948(33.6)	3390(32.8)	1558(32.6)	
District	1706(19.5)	1998(19.4)	690(14.4)	
Clinic	1667(19.0)	2706(26.2)	1106(23.2)	
Urbanization				<0.001
Urban	2820(32.2)	1285(12.4)	1734(36.3)	
Suburban	4188(47.8)	3414(33.1)	2337(48.9)	
Rural	1761(20.1)	5626(54.5)	706(14.8)	

Abbreviation: SES, socioeconomic status; CCIS, Charlson Comorbidity Index Score.

### Univariate survival analysis

The results of our univariate survival analysis indicate that patients with high individual SES have a higher survival rate compared with the group comprising all patients (*p* < 0.001). The DM 4-year survival rates for DM patients with low individual SES living in disadvantaged neighbourhoods were higher than for those living in advantaged neighbourhoods (Table 2).

**Table 2:** Combined effect of individual SES and Neighbourhood SES on 4-Year overall survival rates in diabetes mellitus patients (n = 23,871).

	Total	Case	Survival rate (%)	*P value*
Individual SES				<0.001
Low	8769	1052	85.9	
Moderate	10325	513	94.1	
High	4777	183	95.4	
Neighbourhood SES				<0.001
Disadvantaged	12946	808	92.6	
Advantaged	10925	940	89.8	
Individual*Neighbourhood SES				<0.001
Low SES Disadvantaged	3794	415	87.1	
Low SES Advantaged	4975	637	85.0	
Moderate SES Disadvantaged	7304	323	94.8	
Moderate SES Advantaged	3021	190	92.5	
High SES Disadvantaged	1848	70	95.4	
High SES Advantaged	2929	113	95.4	

**Fig 1:**
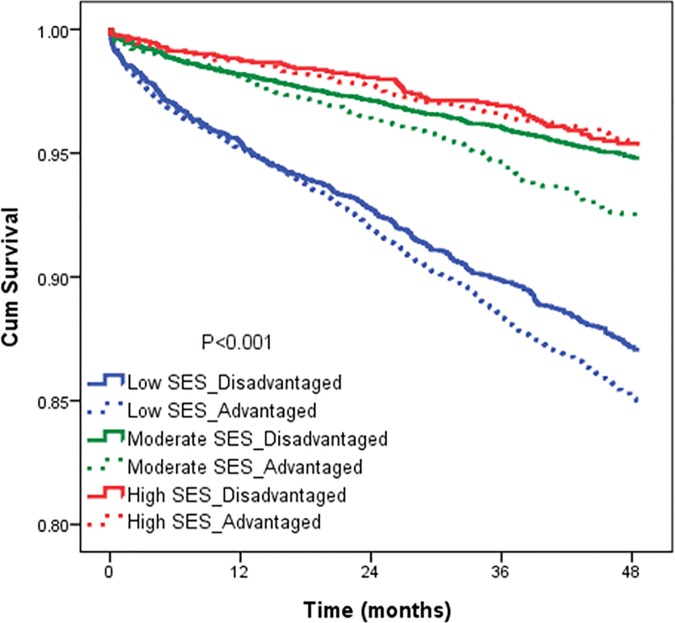
Survival curves by individual-level and Neighbourhood-level SES for DM patients.

Multivariate analysis using cox proportional hazard regression model showed that the combined effects of individual and neighbourhood SES on patient’s survival rates still present significant after adjustment for other factors. After adjusting for age, gender, CCIS, and geographic region, hazard ratios (HRs) reveal that those individuals with low SES living in advantaged neighbourhoods had a 2.43 times higher risk of death (95% confidence interval [CI]: 1.95–3.01) than high SES individuals living in advantaged neighbourhoods. In model 2, they have a 2.46 times higher risk of death (95% CI: 2.07–2.92) than those with high SES individuals living in advantaged neighbourhoods, and an 0.85 times lower risk than high SES individuals living in disadvantaged neighbourhoods. Regression model 3, to which we add further hospital characteristics, teaching level, and urbanization, indicates that DM patients in disadvantaged neighbourhoods with high individual SES had the same risk of mortality as those with high individual SES in advantaged neighbourhoods (HR: 1.11; 95% CI: 0.81–1.51). Patients with low individual SES who lived in disadvantaged areas had higher mortality than those with high SES (odds ratio [OR]: 2.57; 95% CI: 2.04–3.24) (Table 3). The 4-year survival curve is demonstrated in Fig 1.

**Table 3:** Adjusted odds ratios of individual SES and Neighbourhood SES for mortality (n = 23,871).

	Adjust OR*	95% CI	*P value*
Model 1*			
Individual × Neighbourhood SES			
High SES Advantaged	1		
High SES Disadvantaged	1.04	0.76–1.41	0.826
Moderate SES Advantaged	1.45	1.13–1.85	0.003
Moderate SES Disadvantaged	0.85	0.67–1.07	0.164
Low SES Advantaged	2.43	1.95–3.01	<0.001
Low SES Disadvantaged	2.39	1.91–2.99	<0.001
*Model 2*			
Individual SES			
High	1		
Moderate	1.08	0.90-.30	0.417
Low	2.46	2.07–2.92	<0.001
Neighbourhood SES			
Advantaged	1		
Disadvantaged	0.85	0.76–0.95	0.003
Model 3**			
Individual × Neighbourhood SES			
High SES Advantaged	1		
High SES Disadvantaged	1.11	0.81–1.51	0.511
Moderate SES Advantaged	1.50	1.17–1.92	0.001
Moderate SES Disadvantaged	1.06	0.82–1.36	0.658
Low SES Advantaged	2.34	1.89–2.91	<0.001
Low SES Disadvantaged	2.57	2.04–3.24	<0.001

*Model 1 and model 2 adjust for the patients’ age, gender, geographic region, and comorbidities.

**Model 3 adjust for the patients’ age, gender, geographic region, comorbidities, hospital characteristics teaching level and urbanization.

## Discussion

This study found that with DM patients with low individual SES living in disadvantaged neighbourhoods were at a 2.57 times higher risk of mortality than those with high SES living in advantaged neighbourhoods, after adjusting for gender, age, and CCIS. To our knowledge, this study is the first to assess the combined effect of individual and neighbourhood SES in a population-based study of the risk of death using data provided by the national health insurance system.

Although SES has been studied to indicate a significant impact on the survival of other diseases, including head and neck cancer,[23] its role in DM survival has not been valued. A small number of research reports have focused on SES and DM co-morbidity and mortality.[24–26] For example, Walker et al. observed a significant association between low individual SES, greater co-morbidity, and a high mortality rate. Lee et al. found that individuals with low SES have a higher incidence and prevalence rates for DM.[27, 28] However, neither report investigated whether both individual and neighbourhood SES contribute to DM survival rates.

Neighbourhood features that may affect DM survival can be physical or social characteristics of the neighbourhood environment. Whether a patient lives in an advantaged or disadvantaged community may influence the accessibility of medical resources or the frequency with which patients undertake beneficial behaviours that affect the DM survival rate. Among the DM patients in our study, those with low individual SES had the highest risk of mortality, regardless of whether they lived in an advantaged or a disadvantaged neighbourhood. Patients in the low individual SES group tended to live in urban and suburban areas or live in northern and southern Taiwan, and to undergo more frequent treatment in medical centres and regional hospitals, which suggests that they may have undertaken insufficient physical activity and paid inadequate attention to an appropriate diet, such that they were exposed to behavioural and environmental risk factors that reduced their survival rates. Studies have indicated that less physical activity increases the risk of diabetes-related deaths, and that more physical activity improves survival rates in patients with diabetes.[29] In addition, modification of the diet to, e.g., a low-fat, low-salt, or low-carbohydrate Mediterranean diet has been found to be useful in preventing the development and progression of DM.[30–32]

The competitive cause of death, i.e., the fact that complications, rather than DM itself, are often the cause of death, may be the reason for the inverse relationship found between the effects of individual SES and neighbourhood SES on DM survival. Patients with diabetes often also have cardiovascular disease, atherosclerosis, and diabetic nephropathy.[33] Usually, complications are the primary cause of death and the time of diabetes onset is much earlier. As a result, the initial data showed the opposite relationship to our final results, and it was only after correction by the regression models that our results indicated that patients with high individual SES have a lower mortality rate, but that the results are not significant for neighbourhood SES.

Our study did not find a significant correlation between lower neighbourhood SES and the mortality rate in DM patients after adjusting for hospital characteristics, including teaching level and urbanization. Similarly, Millstein et al. study also found no significant relationship between these two variables despite the worse dietary patterns and body-mass index results among low-income and urban African American populations.[34] The possible reasons for the above findings among this group may be caused by their increased competing mortality and food environments.

Our study evaluating the effects of individual and neighbourhood SES on mortality in patients with diabetes mellitus results emphasize the need better treatment information and get more health education and treatment, improved service availability, and more additional social support, regardless of whether they reside in advantaged or disadvantaged neighbourhoods.

In addition, doctors in treatment of patients with diabetes should be to understand the impact of SES on clinical outcomes, particularly those with low SES individual, which can improve the survival rate by improving the accessibility and availability of medical care.

One limitation of our study is that the diagnosis of DM and of any co-morbidity was obtained from ICD codes on National Health Insurance claims. Although it is not its role, so in Taiwan’s National Health Insurance Bureau, a random check charts and interviews with some patients to confirm the accuracy of the diagnosis. Another issue is unable to obtain detailed information from database of insurance claims database related to dietary patterns, smoking habits, body mass index, and other risk factors that may affect the DM survival, such as alcohol consumption. Therefore, the future study should be designed to review DM mortality and the survival rate of these different variables to garner more information on the effect through lifestyle questionnaires or dietary frequency questionnaires. However, this study given the demonstrated soundness of the statistical analysis, these restrictions do not compromise the validity of our study results.

In summary, our research paper is the first independent and combined effects of individual and neighbourhood SES is linked to the 4-year overall survival rate of patients with diabetes. Our finding that there is a high risk of mortality among DM patients with low individual SES, regardless of whether they live in an advantaged or a disadvantaged neighbourhood, suggests that public health attempts to address the existing divergence in SES between DM patients and to manage their disease based on the SES of the patients should be encouraged.

## Abbreviations

SES: socioeconomic status
HR: hazard ratio
CI: confidence interval
OR: odds ratio
SCC: squamous cell carcinoma
ICD-9-CM: International Classification of disease, Ninth Revision, Clinical Modification
CCIS: Charlson Comorbidity Index Score
EC: enrollee category
